# Associations between tooth agenesis and displaced maxillary canines: a cross-sectional radiographic study

**DOI:** 10.1186/s40510-018-0226-0

**Published:** 2018-07-20

**Authors:** Giuseppina Laganà, Nicolò Venza, Roberta Lione, Carlo Chiaramonte, Carlotta Danesi, Paola Cozza

**Affiliations:** 1 Department of Clinical Sciences and Translational Medicine, University of Rome Tor Vergata, Rome, Italy; 2 Department of Statistics, University Hospital of Rome Tor Vergata, Rome, Italy

**Keywords:** Hypodontia, Displaced maxillary canine, Panoramic radiograph, Guidance theory

## Abstract

**Background:**

The aim of present study was to test the association between hypodontia and displaced maxillary canine when compared with a control group.

**Methods:**

The study group was composed of 336 subjects with a mean age of 10.7 ± 1.2 years, presenting with at least one missing tooth. Exclusion criteria included syndromes, craniofacial malformations, extractions and trauma history and previous orthodontic treatment. The control group consisted of 336 subjects with a mean age of 10.7 ± 1.2 years, without agenesis. Stepwise multiple logistic regression using the backwards elimination and the Wald test method was performed to identify the best combination of hypodontia and displaced maxillary canine (*P* < 0.05).

**Results:**

The most represented category in study group consisted in mild agenesis (86.9%); moderate and severe agenesis groups respectively represented the 11.7 and 1.4% of study group. Agenesis was diagnosed in both arches in 46 subjects. Maxillary hypodontia and mandibular hypodontia was respectively detected in 156 and 134 subjects. The most frequent missing teeth were mandibular second premolars (45.8%), lateral incisors (41.7%) and maxillary second premolars (17.8%). A significant correlation between agenesis and displaced maxillary canine was observed in the study group (*P* < 0.05). Only the agenesis of maxillary lateral incisors remained in the final model during backward stepwise deletion. Significant association between the severity of dental agenesis and prevalence of displaced maxillary canine was not assessed.

**Conclusions:**

The outcomes revealed no difference related to the severity of dental agenesis and prevalence of displaced maxillary canine. Only the agenesis of maxillary lateral incisors should be considered directly connected with displaced maxillary canine.

**Electronic supplementary material:**

The online version of this article (10.1186/s40510-018-0226-0) contains supplementary material, which is available to authorized users.

## Background

Congenital missing teeth, or hypodontia, refer to the lack of development of one or more teeth in the primary dentition, and/or in the permanent dentition [1, 2]. In the primary dentition, the agenesis has been found to be less frequent being between 0.1 and 2.4% and it is usually followed by permanent tooth missing. The prevalence of agenesis in the permanent dentition, excluding the third molars, ranges between 0.15 and 16.2% [3] with a higher prevalence in females than males [4–6].

Teeth that erupt in critical terminal areas of the dental lamina (such as the upper lateral incisor, second premolars, third molars) and those located in the embryonic fusion areas are most frequently affected by agenesis, following the so-called end of series [7].

Two forms of hypodontia have been described: syndromic hypodontia refers to tooth agenesis in subjects who have an underlying recognizable clinical syndrome [8] while the non-syndromic (or familiar) hypodontia is the most common form and different inheritance modes were found for this condition [9, 10].

Clinically, hypodontia of the lateral incisors is often observed in association with displacement of maxillary canines (DMC), a condition in which a maxillary canine does not follow its normal eruption path. The prevalence of maxillary canine impaction reaches 1 to 5% [11–13]. Recently, several studies aimed at identifying specific and nonspecific etiological factors or causative genes that can explain the underlying mechanisms involved in the impaction and eruption of maxillary canines [14]. Two hypotheses related to the etiology of DMC have been proposed. The first one suggests that when the lateral incisor is absent or abnormal, the canine will not find the guidance that would enable it to descend along its normal eruption path [15]. A 3-dimensional study, conducted by Kim [16] on 89 CT images of subjects with DMC, indicated that the root and the crown of the maxillary lateral incisor play a key role in the eruption of the maxillary canine. The second hypothesis states that abnormal maxillary canine eruption is genetically determined. Svinhufvud and coworkers in 1988, in a family study of 406 children that received orthodontic treatment, suggest a common genetic origin for DMC and hypodontia [17]. According to this theory, Peck in 2009 included hypodontia and DMC in the Dental Anomaly Pattern which comprises tooth agenesis, microform teeth, delayed tooth development, palatal displaced maxillary canines, infraocclusion of deciduous molars, and mandibular second premolar distal angulation [18]. The correlations between PDC and dentoskeletal characteristics in the sagittal plane (molar relationships and sagittal maxillomandibular discrepancy) have been studied in the past [19, 20]. The literature does not provide information regarding the skeletal relationships in association with PDC but an increased prevalence of III class craniofacial patterns and an occlusal deep bite characteristic has been described in PDC subjects [21, 22].

However, no consensus has been reached as regards the genetic association of dental anomalies, and the genes responsible for DMC have not yet been reached. For this reasons, the DMC’s etiology is still a controversial topic. Pirinen et al. [23] analyzed the pattern of hypodontia on 106 subjects who had had surgical and orthodontic treatment for DMC. Incisor-premolar hypodontia and peg-shaped incisors were found to be strongly associated with DMC [23]. Jang et al. [24], in a radiographic study conducted on 187 patients with DMC, observed a significant association between DMC and maxillary second premolar and lateral incisor agenesis. Considering this background, epidemiological and cross-sectional studies on a large sample might reveal more information about the phenomena. The aims of the present study were to (1) investigate prevalence, characteristics, sex distribution, and significant associations with DMC in a group of growing subjects affected by tooth agenesis; (2) analyze whether the severity (mild, moderate, or severe) of the tooth agenesis has an effect on dental development and presence of DMC; and (3) evaluate whether hypodontia can be a factor involved in maxillary canine impaction when isolated in the maxilla or in the mandible.

The null hypotheses for this study were that (1) there is no significant difference about occurrence of canine displacement in subjects with dental agenesis compared with a control group and (2) hypodontia does not have a role in maxillary canine impaction.

## Methods

An initial sample of digital panoramic radiographs (DPR) of 4706 Caucasian subjects aged between 9 and 12 years, with no genetic syndromes or craniofacial malformations (e.g., cleft lip/palate) and no history of extraction, trauma, or previous orthodontic treatment drawn from the files of the Department of Orthodontics of the University of Rome “Tor Vergata”, was examined. All DPRs were collected from January 2006 to July 2015 and presented with a good quality to allow the assessment of crown and root development. The study project was approved by the Ethic Committee at the University of Rome “Tor Vergata”, and written consent was obtained from all subjects’ parents.

From the initial sample of 4706 DPRs, 325 DPRs of subjects (161 females, 164 males) with a mean age of 11.3 ± 1.2 years were selected; the inclusion criteria considered for the study group (SG) were at least one missing tooth (excluding the third molars) and root development of maxillary canines nearly completed (9: root almost completed, 10: root completed) [25]. The diagnosis of tooth agenesis was based on when no sign of crown calcification and no evidence of loss attributable to orthodontic treatment, caries, periodontal disease, or trauma were found [26].

The SG was divided into three subgroups according to agenesis severity: mild agenesis with 1–2 absent teeth, moderate agenesis with 3–5 absent teeth, and severe agenesis or oligodontia with 6 or more absent teeth [27]. The control group (CG) consisted of 325 (1:1 ratio) subjects, selected from the same initial sample of 4706 DPRs. The CG matched the SG as to gender and stage of the dentition.

After the selection of both SG and CG, using the method described by Ericson and Kurol [28] on the panoramic radiograph to assess DMC, the following linear and angular measurements were measured for each subject of the study and control groups:

- Alpha angle defined as the angle formed by the long axis of the canine and the midline;
- *d* distance defined as the distance in millimeters from the canine cusp tip to the occlusal plane;
- Sector: mesiodistal crown position in sector 1–5.

As previously described by Naoumova and Kjellberg [29], DMC was assessed in both groups only when all the conditions for severe DMC were present: an alpha angle ≥ 30°, *d* distance ≥ 15 mm, canine tip cusp in sector 4 or 5.

All images were evaluated independently by two different operators (N.V. and G.L.) on a computer monitor in a quiet room with subdued ambient lighting. If there was disagreement between the investigators, consensus was reached after discussion.

### Statistical analysis

The data were analyzed by using SPSS software package (Statistical Package for Social Sciences, version 16.0, SPSS Inc., Chicago, USA). Descriptive statistics were used to describe both sample groups (SG and CG) in terms of age, sex, and prevalence rate of missing teeth.

The associations between gender distribution, location in the arch, severity of agenesis, and DMC were analyzed by chi-square test. DPRs in the SG and CG were compared for the prevalence rate of DMC using odds ratio. Stepwise multiple logistic regression using the backwards elimination and the Wald (W) test method was performed to identify the best combination of DMC and dental agenesis. Backward logistic regression is desirable when multiple parameters that are correlated are combined. In backward stepwise logistic regression, all the independent variables are initially entered into the regression equation. The impact of the removal of the single independent variable that reduces the variables by the smallest amount is ascertained. If the decrease is not statistically significant, that variable is permanently eliminated. The process is continued until all remaining variables are significant. This process minimizes the interaction between independent variables and therefore identifies the best combination of variables for predicting the dependent factor or outcome. The level of *P* < 0.05 was considered statistically significant.

## Results

The SG aged between 10.7 and 11.8 years has a mean age of 11.3 years and SD of 1.2 years. The sample sizes and gender distribution are presented in Table 1. The male/female ratio of dental agenesis in both groups was about 1:1. In the SG, the largest category was mild agenesis with 284 subjects (87.4%); moderate and severe agenesis were observed in 36 subjects (11.0%) and in 5 subjects (1.5%), respectively. Significant association between the number of missing teeth and prevalence of DMC was not assessed.

**Table 1:** Descriptive analysis of the sample

	*N*°	Age (years)	SD (years)	Mild H	Moderate H	Severe H
Study group (SG)						
M	164	11.0	± 1.2	145	20	2
F	161	11.6	± 1.3	139	16	3
TOT	325	11.3	± 1.2	284 (87.4%)	36 (11.0%)	5 (1.5%)
Control group (CG)						
M	164	11.2	± 1.2			
F	161	11.4	± 1.3			
TOT	325	11.3	± 1.2			

*M* male, *F* female, *SD* standard deviation, *H* hypodontia

The most frequent missing teeth were mandibular second premolars (*n* = 148; 45.5%), maxillary lateral incisors (*n* = 134; 41.2%), and maxillary second premolars (*n* = 60; 18.4%) (Table 3). The overall prevalence of DMC was higher in the SG than in CG, respectively, 13.5% (*n* = 44) and 5.2% (*n* = 17) with an odds ratio value of 1139 and *P* value of 0.0004 (Table 2).

**Table 2:** Distribution of displacement of maxillary canine in subjects with tooth agenesis compared with control group

	Study group	Control group	*Z* Gauss	*P* value	Standard deviation	Odds ratio	CI 95%
DMC	13.5%	5.2%	3.158	0.0004^a^	0.287	1.139	1.43–4.15

*DMC* displacement of maxillary canines

^a^Sig < 0.05, stepwise multivariate logistic regression

Agenesis diagnosed in the two arches registered for 13.8% (*n* = 45). Maxillary hypodontia was observed in 151 subjects and mandibular hypodontia was detected in 129 subjects. The prevalence of DMC in the maxillary hypodontia group (14.8%) was higher than that in the mandibular hypodontia group (9.6%), with a chi-square value of 1.679 and *P* value of 0.192.

After the stepwise selection process, during which 11 different models were assessed, only one variable was left in the final model (Mx.I2 agenesis) as shown in Table 3, which lists the regression coefficients, standard error, Wald index, degree of freedom, statistical significance, odds ratio, and confidence interval of variable in the final model. Agenesis of maxillary first premolars (Mx.P1), maxillary second premolars (Mx.P2), maxillary second molars (Mx.M2), mandibular first incisors (Mn.I1), mandibular second incisors (Mn.I2), mandibular first premolars (Mn.P1), mandibular second premolars (Mn.P2), and mandibular second molars (MnM2) was dropped from the model during backward stepwise deletion.

**Table 3:** Distribution of missing teeth and statistical association with displacement of maxillary canine

	*P* (%)	*β*	SE	*W*	DF	*S*	OR	CI 95%
Variable in the final model								
Mx.I2 agenesis	41.2	2.174	0.307	28.899	1	0.000^a^	9.243	4.070–20.301
Variables removed during backward stepwise deletion								
Mx.P1 agenesis	13.8							
Mx.P2 agenesis	18.4							
Mx.M2 agenesis	2.4							
Mn.I1 agenesis	1.5							
Mn.I2 agenesis	2.4							
Mn.P1 agenesis	2.4							
Mn.P2 agenesis	45.5							
Mn.M2 agenesis	5.2							

*P* prevalence, *β* regression coefficients, *SE* standard error, *W* Wald index, *DF* degree of freedom, *S* statistical significance, *OR* odds ratio, *CI* confidence interval, *Mx*.*I2* maxillary second incisors, *Mx*.*P1* maxillary first premolars, *Mx*.*P2* maxillary second premolars, *Mx.M2* maxillary second molars, *Mn.I1* mandibular first incisors, *Mn.I2* mandibular second incisors, *Mn.P1* mandibular first premolars, *Mn.P2* mandibular second premolars, *Mn.M2* mandibular second molars

^a^Sig < 0.05, stepwise multivariate logistic regression

Analysis showed DMC is significantly associated with the agenesis of maxillary lateral incisors, whereas the mandibular second premolar or other types of agenesis did not show any significant association (Table 3).

## Discussion

The present retrospective epidemiological study focused on the possible associations between tooth agenesis and DMC in a large sample of Caucasian subjects, when compared with a CG of subjects without agenesis (Additional files 1 and 2).

The chronological age in this type of investigations is a crucial factor as it directly affects the detection of DMC [30]. Previous studies observed that when the lateral incisor is not yet fully developed, panoramic radiographs more commonly show overlapping of the canine and lateral incisor. Thus, when lateral incisor root development is almost complete and overlapping of canine with lateral incisor is observed, a greater mesial inclination of the canine is present [31]. Moreover, hypodontia and DMC are often associated with delayed dental development [27, 32]. For these reasons, in order to minimize false-positive results, it was decided to use root development of maxillary canines nearly completed according to the root development stage method described by Nolla, as inclusion criteria for both SG and CG. To our knowledge, no previous studies analyzed the correlation between tooth agenesis and DMC in a so large group of 325 growing subjects with at least one missing tooth, selected by means of DPR, and compared with a CG derived from the same initial sample.

Garib et al. [33] examined a sample of 126 patients with agenesis to reveal the pattern of associations among DMC and other dental anomalies. Al-Abdallah [34] used a sample of 106 subjects with maxillary hypodontia and 70 with mandibular hypodontia to evaluate the association between DMC and other dental anomalies in an orthodontic population.

The current study found that mild hypodontia was the most common form (87.4%) followed by moderate hypodontia (11.0%), and finally followed by severe hypodontia (1.5%). Mandibular second premolars (45.5%), maxillary lateral incisors (41.2%), and maxillary second premolars (18.4%) resulted as the most frequently missing teeth.

These findings are in agreement with a recent systematic review conducted by Khalaf and co-workers [4] that registered similar prevalence for mild, moderate, and severe hypodontia and a similar distribution for most affected teeth analyzing a total of 93 studies carried out between 1936 and 2012.

Several studies reported a prevalence of DMC between 3 and 5%; according to this, the prevalence of DMC in the CG of our study was 5.2%. Moreover, an increasing of DMC in SG (13.5%, *P* value < 0.05) was observed. A general consensus has been reached in finding that there exists a significant correlation between tooth agenesis and DMC. Sacerdoti and Baccetti [12] reported an increased prevalence of DMC in a sample of subjects with maxillary lateral incisor agenesis, compared with a control group. Moreover, Camilleri [35] observed 106 subjects with DMC and stated a strong connection with hypodontia.

The most important findings of this study were about the association between tooth agenesis and DMC: it was statistically attributable only at the lack of maxillary lateral incisors. Stepwise multiple logistic regression by the backwards elimination and the Wald test method dropped from the initial model all variables except for agenesis of maxillary lateral incisors whereas the mandibular second premolar or other types of agenesis did not show any significant association. The presence of a substantial relationship between agenesis of maxillary lateral incisors and DMC could be explained by the guidance theory. If the lateral incisor is absent, the canine will not find the guidance that would enable it to descend along its normal eruption path and move down in a more palatal path until it comes close to the periosteum of the medial aspect of the alveolar process [36].

According to our results, several studies reported that DMC and agenesis of lateral incisors could be a strong predictor of maxillary canine impaction [37–39]. Jang and coworkers [24] described similar findings of higher correlation with maxillary lateral incisor agenesis and DMC, enrolling 187 cases of DMC and comparing them with a control group. They also report that the correlation between DMC and agenesis of the mandibular second premolar did not reach statistical significance [24].

In contrast, Peck et al. [40] found a strong association of DMC with third molar agenesis and second premolar agenesis, whereas upper lateral incisor agenesis was not significantly interrelated.

Furthermore, Garib et al. [41] observed that 21% of the patients with second premolar agenesis had other permanent teeth missing, excluding the third molars, and an increased prevalence of DMC compared with the general population.

Discrepancies among studies may be also due to sample size or selection, regional ethnic population, genetic variability, environmental factors, and different diagnostic evaluating methods of DMC. The same-race contemporary population composed both groups of our study allowing a reliable genetic association evaluation.

The definition based on alpha angle, *d* distance and displaced sectors [28], used in our study, is the most widely applied diagnostic criterion for determining canine displacement. Moreover, the large sample of our study was advantageous in assessing a significant difference about a specific trend of a certain type of agenesis.

## Conclusions

- Significant association between the severity of dental agenesis and prevalence of DMC was not assessed. Mild hypodontia was the most common form (87.4%) followed by moderate hypodontia (11.0%), and finally followed by severe hypodontia (1.5%). Mandibular second premolars (45.5%), maxillary lateral incisors (41.2%), and maxillary second premolars (18.4%) resulted as the most frequently missing teeth.
- Increasing of DMC (respectively, 13.5% in SG and 5.2% in CG, *P* value < 0.05) was observed in SG group compared with CG.
- The association between tooth agenesis and DMC was statistically attributable only at the lack of maxillary lateral incisors. Stepwise multiple logistic regressions dropped from the initial model all variables except for agenesis of maxillary lateral incisors whereas the mandibular second premolar or other types of agenesis did not show any significant association.
- The clinician has to be aware that the absence of maxillary lateral incisors may be considered an early diagnostic marker of the development of DMC.

## Additional files

Additional file 1: Control Group (subjects without agenesis). (PDF 60 kb)

Additional file 2: Study Group (subjects with at least one missing tooth). (PDF 71 kb)

## Abbreviations

CG: Control group
DMC: Displacement of maxillary canines
DPR: Digital panoramic radiographs
Mn.I1: Mandibular first incisors
Mn.I2: Mandibular second incisors
Mn.M2: Mandibular second molars
Mn.P1: Mandibular first premolars
Mn.P2: Mandibular second premolars
Mx.I2: Maxillary second incisors
Mx.M2: Maxillary second molars
Mx.P1: Maxillary first premolars
Mx.P2: Maxillary second premolars
SG: Study group
