# Supplementary material for: Associations between tooth agenesis and displaced maxillary canines: a cross-sectional radiographic study
Source: Prog Orthod. 2018 Jul 20;19:23. doi: 10.1186/s40510-018-0226-0 (PMC6053345; doi:10.1186/s40510-018-0226-0)
Supplement: Supplementary file 1 — Control Group (subjects without agenesis). (PDF 60 kb) [file 40510_2018_226_MOESM1_ESM.pdf]

cod	SEX	BIRTH	AGE	OP TAKEN	malposizione CANINO SUP DX	malposizione CANINO SUP SX	dentatura permanente
					DMC-D	DMC-S	DP
85112	M	3/7/01	8,94	10/6/10	0	0	0
84761	M	21/8/01	8,77	28/5/10	0	0	0
84094	M	4/4/02	8,10	10/5/10	0	0	0
83781	M	16/7/02	7,79	30/4/10	0	0	0
83679	M	10/1/02	8,30	28/4/10	0	0	0
82621	M	29/1/02	8,17	31/3/10	0	0	0
80508	M	7/3/02	7,92	3/2/10	0	0	0
80150	M	20/2/02	7,94	26/1/10	0	0	0
79383	M	20/5/01	8,64	5/1/10	0	0	0
70261	M	16/7/02	8,38	30/11/10	0	0	0
77879	M	4/5/01	8,54	14/11/09	0	0	0
77491	M	21/1/02	7,82	15/11/09	0	0	0
76953	M	20/3/01	8,76	20/12/09	0	0	0
77356	M	20/2/02	7,86	28/12/09	0	0	0
75076	M	8/11/00	8,62	21/6/09	0	0	0
65818	M	21/6/02	8,58	18/1/11	0	0	0
76437	M	5/12/01	7,97	23/11/09	0	0	0
76161	M	19/4/01	8,47	7/10/09	0	0	0
76114	M	6/8/01	8,18	10/10/09	0	0	0
76050	M	1/12/01	7,85	7/10/09	0	0	0
75934	M	2/2/01	8,67	2/10/09	0	0	0
75924	M	1/3/01	8,61	6/10/09	0	0	0
75757	M	1/11/01	7,96	15/10/09	0	0	0
74555	M	13/2/01	8,54	27/8/09	0	0	0
72750	M	29/1/01	8,39	18/6/09	0	0	0
71742	M	21/1/01	8,26	25/4/09	0	0	0
64609	M	8/9/02	8,47	24/2/11	0	0	0
71243	M	6/1/01	8,29	21/4/09	0	0	0
48143	M	22/8/99	7,87	3/7/07	0	0	0
50032	M	26/1/99	8,61	3/9/07	0	0	0
64599	M	26/1/00	8,75	22/10/08	0	0	0
64352	M	27/3/00	8,56	17/10/08	0	0	0
64301	M	29/7/00	8,20	7/10/08	0	0	0
57125	M	25/1/01	8,05	12/2/09	0	0	0
62073	M	20/9/00	7,84	23/7/08	0	0	0
61064	M	13/5/00	8,11	21/6/08	0	0	0
45952	M	6/5/00	8,32	30/8/08	1	0	0
52541	M	11/1/00	7,80	27/10/07	0	0	0
51532	M	7/1/99	8,74	2/10/07	0	0	0
52128	M	21/4/99	8,48	13/10/07	0	0	0
123896	M	6/12/98	8,78	14/9/07	0	0	0
130595	M	12/04/07	8,16	09/06/15	0	0	0
96867	M	12/12/05	8,75	09/09/14	0	0	0
114919	M	01/03/05	8,89	17/01/14	0	0	0
118867	M	22/11/05	8,26	22/02/14	0	0	0
118524	M	30/03/04	8,92	26/02/13	0	0	0
121604	M	25/02/05	8,22	15/05/13	0	0	0
113927	M	09/03/05	8,51	10/09/13	0	0	0
113651	M	27/01/05	8,26	29/04/13	0	0	0
48549	M	17/07/05	7,83	13/05/13	0	0	0
111124	M	30/10/04	8,33	26/02/13	0	0	0
109363	M	16/03/04	8,83	11/01/13	0	0	0
110003	M	05/06/04	8,30	19/09/12	0	0	0
109174	M	11/11/04	7,85	17/09/12	0	0	0
108793	M	28/08/04	8,04	10/09/12	0	1	0
109280	M	20/02/04	8,56	11/09/12	0	0	0
107729	M	16/11/03	8,64	05/07/12	0	0	0
107000	M	22/06/04	7,98	11/06/12	0	0	0
106891	M	22/01/04	8,40	14/06/12	0	0	0
106854	M	02/06/04	8,04	13/06/12	0	0	0
82560	M	18/7/00	9,70	30/3/10	1	0	0
71486	M	10/1/02	9,21	24/3/11	0	0	0
79025	M	3/7/01	9,37	14/11/10	0	0	0
77152	M	15/9/00	9,25	15/12/09	0	0	0
63037	M	29/9/01	9,08	26/10/10	0	0	0
60786	M	1/3/01	9,74	25/11/10	0	0	0
55570	M	6/5/00	9,81	23/2/10	0	0	0
74923	M	15/1/00	9,67	15/9/09	0	0	0

72061	M	14/6/99	9,92	12/5/09	0	0	0
72021	M	7/9/99	9,68	11/5/09	0	0	0
71378	M	14/11/99	9,45	26/4/09	0	0	0
29008	M	26/7/98	9,10	29/8/07	0	0	0
68805	M	13/12/99	9,96	26/11/09	0	1	0
65719	M	14/11/99	9,01	14/11/08	0	0	0
64871	M	26/1/02	9,75	26/10/11	0	0	0
64673	M	15/1/99	9,76	18/10/08	0	0	0
64652	M	25/3/02	9,57	17/10/11	0	0	0
53070	M	28/9/99	9,34	27/1/09	0	0	0
63736	M	11/8/02	9,26	11/11/11	0	0	0
63632	M	7/1/02	9,75	5/10/11	0	0	0
62722	M	21/11/01	9,90	14/10/11	0	0	0
62486	M	30/1/99	9,80	14/11/08	0	0	0
51753	M	18/8/99	9,19	24/10/08	1	1	0
51095	M	7/8/99	9,25	3/11/08	0	0	0
24098	M	29/5/99	9,40	18/10/08	0	0	0
53172	M	24/12/01	9,90	14/11/11	0	0	0
53023	M	20/4/98	9,60	22/11/07	0	0	0
18782	M	3/10/97	9,91	30/8/07	0	0	0
50091	M	12/11/97	9,80	30/8/07	0	0	0
49806	M	8/3/98	9,50	6/9/07	0	0	0
106545	M	24/11/04	9,30	10/03/14	0	0	0
130264	M	20/05/05	9,91	14/04/15	1	0	0
129883	M	12/11/05	9,45	23/04/15	0	0	0
129186	M	21/01/06	9,06	09/02/15	0	0	0
128983	M	03/03/05	9,89	21/01/15	1	1	0
112458	M	23/05/05	9,68	23/01/15	0	0	0
120428	M	15/09/04	9,23	05/12/13	0	0	0
115249	M	21/02/04	9,78	28/11/13	0	0	0
119868	M	16/02/04	9,17	15/04/13	0	0	0
115869	M	03/07/04	9,29	15/10/13	0	0	0
117960	M	09/03/04	9,22	27/05/13	0	0	0
116500	M	26/07/03	9,68	28/03/13	0	0	0
116494	M	01/03/04	9,08	28/03/13	0	0	0
115655	M	20/07/03	9,69	26/03/13	0	0	0
106891	M	17/12/03	9,92	14/11/13	0	0	0
104628	M	09/02/04	9,71	23/10/13	0	0	0
103477	M	22/04/04	9,49	15/10/13	0	0	0
110841	M	27/08/03	9,18	27/10/12	0	0	0
112645	M	20/12/02	9,93	23/11/12	0	0	0
110580	M	14/08/03	9,17	12/10/12	0	0	0
106980	M	05/02/03	9,35	11/06/12	0	0	0
84933	M	2/12/99	10,51	3/6/10	0	0	0
82439	M	20/1/00	10,18	25/3/10	1	0	0
65108	M	8/1/00	10,79	20/10/10	1	1	0
77848	M	3/11/99	10,07	24/11/09	0	0	0
76573	M	1/3/99	10,29	12/6/09	0	0	0
59439	M	3/8/99	10,22	21/10/09	0	0	0
76379	M	15/9/99	10,08	12/10/09	0	0	0
73327	M	29/5/99	10,06	17/6/09	0	0	0
49085	M	6/9/96	10,83	3/7/07	0	0	0
64042	M	29/5/01	10,35	3/10/11	0	0	0
63631	M	10/5/01	10,41	6/10/11	0	0	0
62038	M	2/2/98	10,47	21/7/08	0	0	0
35076	M	13/8/98	10,14	29/9/08	0	0	0
40543	M	6/5/97	10,75	3/2/08	0	0	0
28001	M	1/11/97	10,31	19/2/08	0	0	0
51095	M	11/11/96	10,89	29/9/07	0	0	0
106800	M	31/05/04	10,86	08/04/15	0	0	0
117852	M	13/10/02	10,89	30/08/13	0	0	0
127203	M	22/11/03	10,85	24/09/14	0	0	0
79411	M	10/11/03	10,87	19/09/14	0	0	0
111884	M	06/10/03	10,75	02/07/14	0	0	0
117999	M	20/04/02	10,82	09/02/13	0	0	0
103523	M	06/03/03	10,95	13/02/14	0	0	0
101865	M	23/10/03	10,25	21/01/14	0	1	0
112049	M	12/04/02	10,68	12/12/12	0	0	0
109700	M	01/12/01	10,83	27/09/12	0	0	0
109028	M	06/06/02	10,26	06/09/12	0	0	0
107367	M	11/06/02	10,05	28/06/12	0	0	0
19188	M	29/10/90	11,32	18/2/02	0	0	1

84207	M	27/5/98	11,97	12/5/10	0	0	1
83628	M	16/8/98	11,70	27/4/10	0	0	1
83382	M	30/3/99	11,07	21/4/10	0	0	0
82551	M	8/1/99	11,23	29/3/10	0	0	0
52709	M	30/12/98	11,15	19/2/10	0	0	0
76037	M	23/12/97	11,79	6/10/09	0	1	0
72059	M	16/6/97	11,91	12/5/09	0	0	1
50688	M	27/11/95	11,77	1/9/07	0	0	0
61578	M	8/6/97	11,03	15/6/08	0	0	1
61322	M	4/7/96	11,96	16/6/08	0	1	0
52043	M	2/8/96	11,22	18/10/07	0	0	0
14485	M	10/2/96	11,75	8/11/07	0	0	0
50513	M	1/6/96	11,39	18/10/07	0	0	0
24902	M	2/6/96	11,27	6/9/07	0	0	0
98853	M	02/03/02	11,76	30/11/13	0	0	1
127694	M	12/02/02	11,61	18/09/13	0	0	0
126724	M	02/07/03	11,22	18/09/14	0	0	0
112383	M	12/10/03	11,11	17/11/14	0	0	0
120310	M	12/02/02	11,01	13/02/13	0	0	0
95022	M	08/08/02	11,25	06/11/13	0	0	0
92427	M	10/11/01	11,93	12/10/13	0	0	0
112459	M	04/06/01	11,62	14/01/13	0	0	1
110557	M	11/07/01	11,26	11/10/12	0	1	0
108785	M	08/02/01	11,59	07/09/12	0	0	0
108555	M	21/04/01	11,25	19/07/12	0	0	0
106788	M	25/03/01	11,22	11/06/12	0	0	0
29999	M	16/12/96	12,20	25/2/09	0	0	1
52131	M	1/10/95	12,01	3/10/07	0	0	1
51757	M	7/5/95	12,41	1/10/07	0	0	0
8871	F	12/2/94	8,20	23/4/02	0	0	0
83946	F	22/8/01	8,71	5/5/10	0	0	0
80092	F	6/1/02	8,06	25/1/10	0	0	0
77847	F	22/9/01	8,15	13/11/09	0	0	0
77032	F	13/4/01	8,76	13/1/10	0	0	0
76445	F	23/4/01	8,58	18/11/09	0	0	0
75622	F	16/7/01	8,22	1/10/09	0	0	0
75497	F	30/1/01	8,66	25/9/09	0	0	0
75467	F	28/1/01	8,67	28/9/09	0	0	0
72555	F	26/10/00	8,59	27/5/09	0	0	0
72126	F	23/4/01	8,06	13/5/09	0	0	0
72064	F	22/8/00	8,73	12/5/09	0	0	0
72005	F	5/1/01	8,35	12/5/09	0	0	0
71986	F	14/1/01	8,33	11/5/09	0	0	0
71860	F	13/1/03	8,25	13/4/11	0	0	0
67918	F	14/12/01	8,30	1/4/10	0	0	0
63291	F	3/5/01	8,83	27/2/10	0	0	0
42084	F	1/9/98	8,35	4/1/07	0	0	0
26335	F	3/11/98	8,76	6/8/07	1	1	0
65546	F	25/10/00	8,05	10/11/08	0	0	0
54735	F	20/5/00	8,70	27/1/09	0	0	0
62001	F	25/7/00	7,98	17/7/08	0	0	0
61212	F	24/5/00	8,06	12/6/08	0	0	0
52126	F	28/6/99	8,33	26/10/07	0	0	0
51543	F	3/11/98	8,92	3/10/07	0	0	0
51539	F	1/3/99	8,59	2/10/07	0	0	0
51909	F	1/4/99	8,52	4/10/07	0	0	0
51751	F	7/10/99	8,01	10/10/07	0	0	0
40829	F	4/9/99	8,42	2/2/08	0	0	0
50635	F	12/1/00	7,99	7/1/08	0	0	0
122959	F	28/08/05	8,70	07/05/14	0	0	0
128783	F	23/02/06	8,90	17/01/15	0	0	0
127926	F	13/07/06	8,33	10/11/14	0	0	0
127752	F	10/05/06	8,34	09/09/14	0	0	0
126959	F	24/09/06	7,95	03/09/14	0	0	0
126160	F	30/06/06	8,22	18/09/14	0	0	0
124511	F	22/11/04	8,50	22/05/13	0	0	0
114914	F	23/12/05	8,05	10/01/14	0	0	0
120829	F	02/06/05	8,46	15/11/13	0	0	0
120437	F	04/07/05	8,25	03/10/13	0	0	0
119397	F	12/04/05	8,84	12/02/14	0	0	0
114251	F	04/08/05	8,26	04/11/13	0	0	0
113541	F	20/01/05	8,46	04/07/13	0	0	0

112832	F	07/02/05	8,04	21/02/13	0	0	0
118053	F	27/05/04	8,94	02/05/13	0	0	0
115707	F	20/04/04	8,94	27/03/13	0	0	0
109282	F	12/07/05	8,27	15/10/13	0	0	0
111630	F	03/05/04	8,57	24/11/12	0	0	0
111215	F	27/09/04	8,56	16/04/13	0	0	0
111170	F	02/01/05	8,28	11/04/13	0	0	0
114065	F	21/05/04	8,63	03/01/13	0	0	0
113098	F	23/06/04	8,50	20/12/12	0	0	0
110214	F	13/07/04	8,37	23/11/12	1	0	0
110404	F	21/10/04	7,97	09/10/12	0	0	0
110077	F	16/01/04	8,72	03/10/12	1	1	0
108795	F	27/07/04	8,13	10/09/12	0	0	0
108527	F	06/02/04	8,99	30/01/13	0	0	0
106938	F	17/11/03	8,57	11/06/12	0	0	0
106909	F	08/05/04	8,11	14/06/12	0	0	0
85030	F	6/10/00	9,68	8/6/10	0	0	0
84528	F	5/2/01	9,29	21/5/10	0	0	0
80444	F	20/6/00	9,63	2/2/10	0	0	0
80148	F	20/6/00	9,61	26/1/10	0	0	0
72602	F	25/10/01	9,41	22/3/11	0	0	0
78831	F	5/8/00	9,35	10/12/09	0	0	0
64352	F	27/3/01	9,62	8/11/10	0	0	0
71743	F	15/11/00	9,20	25/1/10	0	0	0
70261	F	24/10/00	9,24	18/1/10	0	0	0
50822	F	7/9/01	9,47	25/2/11	0	0	0
74835	F	22/6/00	9,28	2/10/09	0	0	0
43019	F	16/7/98	9,01	16/7/07	0	0	0
49407	F	18/3/98	9,32	9/7/07	0	0	0
65758	F	21/8/99	9,24	13/11/08	0	0	0
65193	F	14/6/02	9,38	30/10/11	0	0	0
64857	F	3/1/99	9,75	2/10/08	0	0	0
64731	F	11/11/98	9,95	21/10/08	0	0	0
64654	F	13/5/99	9,45	21/10/08	0	0	0
64464	F	23/6/99	9,29	5/10/08	0	0	0
23717	F	13/5/99	9,80	25/2/09	0	0	0
63841	F	18/9/02	9,10	21/10/11	0	0	0
61912	F	19/9/01	9,86	29/7/11	0	0	0
61497	F	9/3/99	9,29	21/6/08	0	0	0
40021	F	18/4/99	9,45	27/9/08	0	0	0
49350	F	7/12/98	9,15	30/1/08	0	0	0
49770	F	24/3/98	9,48	12/9/07	0	0	0
48665	F	3/8/97	9,76	4/5/07	0	0	0
103349	F	09/06/04	9,75	07/03/14	0	0	0
130451	F	15/06/05	9,91	12/05/15	0	0	0
107011	F	07/11/05	9,33	05/03/15	0	0	0
119220	F	06/03/05	9,82	29/12/14	0	0	0
117607	F	07/04/05	9,84	05/02/15	1	0	0
128745	F	23/12/04	9,97	11/12/14	0	0	0
95375	F	15/03/05	9,54	25/09/14	0	0	0
120028	F	14/06/04	9,32	05/10/13	0	0	0
107438	F	03/09/04	9,11	12/10/13	0	0	0
81834	F	10/01/00	9,82	04/11/09	0	0	0
116575	F	18/01/03	9,97	04/01/13	0	0	0
109600	F	28/09/03	9,13	10/11/12	0	0	0
109090	F	28/01/03	9,63	11/09/12	0	0	0
108853	F	15/07/03	9,16	10/09/12	0	0	0
110856	F	11/08/03	9,20	20/10/12	0	0	0
106937	F	05/05/03	9,11	11/06/12	0	0	0
106787	F	11/02/03	9,34	11/06/12	0	0	0
111005	F	03/01/03	9,79	15/10/12	0	0	0
18531	F	8/6/91	10,75	7/3/02	0	0	0
77838	F	16/8/99	10,28	25/11/09	1	0	0
75892	F	17/7/99	10,23	7/10/09	0	0	0
75116	F	27/10/98	10,90	16/9/09	0	0	0
69662	F	23/1/00	10,24	19/4/10	0	0	0
27135	F	21/4/98	10,90	11/3/09	0	0	0
63776	F	23/6/01	10,38	5/11/11	0	0	0
62674	F	17/8/98	10,19	24/10/08	0	0	0
62365	F	27/1/01	10,48	18/7/11	0	0	0
62043	F	3/11/97	10,74	27/7/08	0	0	0
31975	F	25/2/98	10,04	10/3/08	0	0	0

49851	F	5/3/97	10,51	5/9/07	0	0	0
80565	F	18/09/03	10,52	24/03/14	0	0	0
75574	F	23/07/03	10,82	14/05/14	0	0	0
120569	F	30/05/04	10,61	05/01/15	0	0	0
119326	F	30/10/04	10,12	11/12/14	0	0	0
109528	F	24/03/04	10,64	12/11/14	0	0	0
101649	F	10/12/03	10,72	26/08/14	0	0	0
116010	F	21/08/03	10,61	29/03/14	0	0	0
123244	F	22/04/03	10,77	24/01/14	0	0	0
114406	F	19/09/02	10,35	23/01/13	0	0	0
117866	F	12/07/03	10,41	05/12/13	0	0	0
87331	F	20/02/03	10,99	13/02/14	0	0	0
75893	F	15/03/03	10,55	30/09/13	0	0	0
108810	F	12/09/02	10,00	10/09/12	1	1	0
106910	F	21/12/01	10,49	14/06/12	0	0	0
83271	F	18/9/98	11,59	19/4/10	0	0	0
81554	F	15/4/98	11,89	3/3/10	0	0	0
79973	F	14/2/98	11,94	21/1/10	0	0	1
79354	F	19/8/98	11,39	4/1/10	0	0	1
79095	F	18/9/98	11,26	18/12/09	0	0	0
71796	F	27/11/97	11,39	17/4/09	0	0	1
40786	F	12/10/98	11,50	8/4/10	0	0	0
51124	F	29/2/96	11,48	22/8/07	0	0	0
9710	F	6/4/00	11,41	2/9/11	0	0	0
16666	F	7/12/98	11,14	23/1/10	0	0	0
33362	F	1/10/97	11,45	11/3/09	0	0	1
61685	F	26/2/94	11,90	18/1/06	0	0	0
61247	F	2/3/97	11,30	15/6/08	0	0	0
52124	F	9/1/96	11,81	30/10/07	0	0	0
51762	F	1/9/96	11,09	2/10/07	0	0	0
12059	F	11/10/96	11,15	3/12/07	0	0	0
50142	F	6/9/96	11,58	2/4/08	0	0	0
110860	F	13/03/03	11,16	09/05/14	0	0	0
110256	F	03/07/02	11,69	10/03/14	0	0	0
130765	F	15/01/04	11,24	10/04/15	0	0	0
129919	F	18/03/04	11,08	16/04/15	0	0	0
123242	F	18/04/04	11,19	25/06/15	0	0	0
126940	F	11/11/01	11,84	10/09/13	0	0	0
129623	F	26/09/03	11,39	12/02/15	0	0	0
115818	F	11/04/02	11,79	20/01/14	0	0	0
114761	F	11/05/02	11,56	30/11/13	0	0	0
119318	F	07/02/03	11,01	06/02/14	0	0	0
119272	F	10/03/02	11,64	25/10/13	0	0	0
121718	F	07/04/02	11,52	11/10/13	0	0	0
97743	F	26/03/02	11,59	23/10/13	1	0	0
80470	F	07/10/02	11,09	05/11/13	0	0	0
77818	F	21/09/02	11,13	05/11/13	0	0	0
72925	F	28/06/02	11,25	25/09/13	0	0	0
113584	F	06/12/01	11,20	14/02/13	0	0	0
112348	F	29/07/01	11,39	17/12/12	0	1	0
109094	F	15/11/00	11,83	11/09/12	0	0	0
110467	F	18/03/01	11,59	17/10/12	0	0	0
			9,66				13
